# Hospital Investments and Trustees: A Practical View of Depreciation

**Published:** 1915-06-26

**Authors:** 


### HOSPITAL INVESTMENTS AND TRUSTEES.

### A Practical View of Depreciation.

Nearly every hospital has a nest-egg which is generally the outcome of surplus income plus sums given or devised specifically for endowment. Each one responsible for the financial side of the work of our voluntary hospitals must have, during the last few years, and especially during the past ten months, surveyed the possessions of his institution and watched the general tendencies of the prices of stocks and shares. When one sees savings laboriously put together seemingly melt away, it is a source of some consolation to search for compensating features, and to consider how far the question of depreciation in the prices of securities is an academic one.

### Income not Increment.

It is as easy as it is vain to say, "If we had done so-and-so, we should have been in such-and-such a position"; but we must never lose sight of the undeniable principle that a hospital is an investor, and not a speculator, and that our investments are, as a rule, made with an eye to produce income instead of increment. That is not to say that the best advice is not to be sought when there is money to be invested, and most of our hospitals have connected with them some specially qualified person (frequently there is such a one on the board of management) who is always ready to be consulted.

But there are times when stocks have to be sold: indeed, it is occasionally the case that investments are made with a view to subsequent realisation, as in the case of a fund for rebuilding. Therefore, the question of depreciation is not altogether an academic one. Let us consider the benefits accruing from the enlistment of skilled assistance, not only in purchase, but in the periodical review of lists of securities.

Under the directions of the central funds, hospitals show in their balance-sheets securities at cost price, and in the annual report disclose full particulars, generally arranged in two lists, of investments—one detailing securities held as endowment of beds, or for other trust purposes, and the other representing accumulated savings. We observe from the report of one of the largest London hospitals that the original cost of funds belonging to that institution under the first-named category was £51,374, and these on March 31, 1915, had depreciated in value to £39,262. The same list at a valuation made on the basis of prices on March 31, 1914, represented in cash £43,248. More than one-half of the securities are in India 3 per cent. stock and Consolidated 2½ per cent. stock. In respect of the funds for general purposes, the corresponding depreciations were as follows:—Original cost, £36,347; valuation, March 31, 1914, £31,174; valuation, March 31, 1915, £28,558. Altogether, the invested funds of this hospital have, since they were bought, fallen in value from £87,721 to £67,820. The funds show a shrinkage of 24 per cent. on original cost, and of 9 per cent. during the last year.

Another instance is the generous gift in 1905 by a member of the London Stock Exchange of certain securities to be held in trust for the benefit of specified charities, chiefly hospitals. The value at that time of the investments was £100,216, and they have yielded close on £4,000 annually to the institutions interested. This fund has been handled by experts, the trustees being men of international reputation, and most eminent as financiers. Not more than £6,000 (nominal) is represented by any one stock, and no doubt the business of the trust has been watched and fostered with close personal care. The value of the fund at present prices is £80,447. The fund has fallen in value 19 per cent. since its foundation, and 8 per cent. since April 1914, when the valuation was £87,467. It is not suggested that there was an ordinary want of care in the management of the first fund, but the specialised management of the latter trust appears to have produced better results.

### The Duty of Trustees.

What is the legal duty of trustees regarding investments? The powers of hospitals as to investment in stocks and shares are not generally known. The executive of a hospital that is not incorporated may invest surplus income within the rule of the hospital respecting investment, and, in the absence of a rule, in suitable (not necessarily trustee) securities, at the discretion of the managing body. But, with regard to funds for the endowment of beds or for other trust purposes, unless expressly forbidden by the trust instruments or allowed to go beyond, these must be invested in securities for the time being authorised by law for trustees. An incorporated hospital shall invest either surplus income or funds for endowment within the limits of that part of the constitution relating to such matters. These limits are often beyond the range of stocks mentioned in the Trustee Act, 1893, and the Colonial Stock Act, 1900; but as incorporation has been granted by Parliament, the Privy Council, or the Board of Trade, it is unlikely that any latitude given can be carried to dangerous limits. Frequently an incorporated society is empowered to retain investments in the form in which the same may be bequeathed or given, and, of course, any directions of a legator in respect of the investment of funds for endowment shall be observed, unless it is thought desirable to seek permission from the Court to vary such instructions.

To what extent are trustees obliged to "watch" securities of a trust? Are the trustees clear of responsibility if, in the making of an investment, they have scrupulously observed the statute, and does their duty end at the time of investment? A Scottish Judge, Lord Neaves, says that it does not end there. This is his pronouncement: "The duty of a trustee is not a duty that arises at a single moment of time alone. It is the duty of a trustee to look vigilantly after the investment when it is made, to get the earliest notice of threatened insolvency or deficiency of credit in the party with whom the investment is deposited, and to take the earliest steps of diligence to make the trust estate secure.'' Our English Judges have not put the duty of inspection so high. Lord Justice Cozens Hardy expresses the view that there is no obligation or duty on the part of trustees to make periodical investigations. The liability of a trustee, he thought, ''must proceed on the footing of wilful default, and not upon not making inquiries when he ought to do so.'' But his lordship made the reservation that a duty might arise when there are circumstances which suggest to a reasonable man that the security is in jeopardy.

### A Legal Definition.

The definition of the legal duty as to inspection is, therefore, vague—the statutes say nothing as to revaluation, keeping up the security, or disposing of it when the proper margin of security has disappeared.

The last thing that is to be encouraged is for hospital trustees to fidget and fuss when investments fali, and try to change and get out; but between that extreme and complete indifference there is a great distance. Where expert advice is available, it should from time to time be utilised in a periodical survey of the list of securities, purely from the point of view of the investor. If this is done, rises and falls can be regarded without emotion, courage will be maintained, and patience cultivated.

It is a curious paradox that (in peace times) low prices of stocks coincide with prosperous times and good trade: during the greater part of the last ten years trade has been unusually good. The explanation of this curious feature of finance is that when people have money that they can make use of profitably in business, they do not need to invest in stocks and shares. Though at all times there is a steady flow of investments by trustees, apparently this is not a sufficient set-off against the non-investment of surplus funds by the general body of the community. Arising out of this result we find one consolatory fact. The years of depreciation in values have mostly been years of prosperity in respect of trade. Therefore, if the hospital has been successful in bringing home its usefulness and its needs to those who have profitably employed the opportunities of the fat years, loss through investments may have been made up by acquisition of new endowments and new securities through the surplus income of good years.

Even if no additions to capital have thus been made, at least no stock has been sold, or not so much as would have been sold, in years of trade depression. Then, from the point of view of investment, there has been no loss of income—there may even have been an improvement in this respect as redeemable stocks (such as the Queensland 4 per cent. 1915 stock) have been redeemed at par or converted into a security yielding a higher rate of interest.

Investments respecting surplus savings, subject to any rule of a hospital to the contrary, may be realised at the discretion of the board. Of course, the last thing that a hospital should do is to part with its capital or to borrow money, but there are times when such a course is necessary. How and when can any part of the endowment fund be sold or pledged? Such power, when needed by a board of management, can be obtained only of the Charity Commissioners, whose conditions for granting an order are rather stringent. The Commissioners will agree to a sale or pledging of stock if sufficient securities are deposited with them to accumulate by means of invested dividends, in order to replace, within an agreed period, the investments sold or pledged. The operation, therefore, entails a serious loss of income for a long time, for not only does a hospital lose the interest on the stock disposed of, but also that on the investments set aside to form the sinking fund. It would seem, therefore, that a hospital must exhaust all immediate resources before accepting such an expensive expedient.

### Payment of Legacies Since the War.

Since the outbreak of the war many hospital secretaries have found the task of obtaining payment of legacies far from easy. The difficulty in dealing with stocks and shares has resulted in much delay in realising estates, and executors of wills have often found themselves in the position of being compelled to postpone payments to beneficiaries. In some cases it has been so impossible to realise securities that even the payment of death duties has been a matter of some embarrassment. Of course, after twelve months from the proving of a will, a legatee is entitled to reasonable interest until the legacy is paid; but a complete settlement of accounts is probably preferred. When a charity is entitled to some part of the residue it is very much against the interests of the body participating that securities should be realised (even if it is possible to realise them) at the present low market prices. The best way out of the trouble, whenever it is practicable, is for the legatee to accept suitable securities to an equal value of the legacy.

We have not attempted to deal here in detail with devises of real estate and the circumstances under which a hospital can hold such property, but in considering the foregoing suggestion as to settlement it must not be forgotten that, under the provisions of the Mortmain Act, 1901, land cannot be held by a charity for more than a year without permission for an extended period having been granted by the High Court.

